# A Novel Role for CD55 in Granulocyte Homeostasis and Anti-Bacterial Host Defense

**DOI:** 10.1371/journal.pone.0024431

**Published:** 2011-10-03

**Authors:** Henrike Veninga, Robert M. Hoek, Alex F. de Vos, Alex M. de Bruin, Feng-Qi An, Tom van der Poll, René A. W. van Lier, M. Edward Medof, Jörg Hamann

**Affiliations:** 1 Department of Experimental Immunology, Academic Medical Center, University of Amsterdam, Amsterdam, The Netherlands; 2 Center for Experimental Molecular Medicine, Academic Medical Center, University of Amsterdam, Amsterdam, The Netherlands; 3 Institute of Pathology, Case Western Reserve University, Cleveland, Ohio, United States of America; Centre de Recherche Public de la Santé (CRP-Santé), Luxembourg

## Abstract

**Background:**

In addition to its complement-regulating activity, CD55 is a ligand of the adhesion class G protein-coupled receptor CD97; however, the relevance of this interaction has remained elusive. We previously showed that mice lacking a functional CD97 gene have increased numbers of granulocytes.

**Methodology/Results:**

Here, we demonstrate that CD55-deficient mice display a comparable phenotype with about two-fold more circulating granulocytes in the blood stream, the marginated pool, and the spleen. This granulocytosis was independent of increased complement activity. Augmented numbers of Gr-1-positive cells in cell cycle in the bone marrow indicated a higher granulopoietic activity in mice lacking either CD55 or CD97. Concomitant with the increase in blood granulocyte numbers, *Cd55* ^-/-^ mice challenged with the respiratory pathogen *Streptococcus pneumoniae* developed less bacteremia and died later after infection.

**Conclusions:**

Collectively, these data suggest that complement-independent interaction of CD55 with CD97 is functionally relevant and involved in granulocyte homeostasis and host defense.

## Introduction

Decay-accelerating factor (CD55) is a GPI-anchored molecule on leukocytes, erythrocytes, and serum-exposed stromal cells that accelerates the decay of the complement convertases C3 and C5 [1], [2]. The importance of CD55 for preventing endogenous cells from unwanted complement activation is evident from the phenotype of CD55-deficient mice that develop exaggerated autoimmune reactions in a variety of spontaneous and induced models [3]–[8]. Furthermore, studies in CD55^-/-^ mice showed that complement activation not only facilitates innate immune responses but also adaptive immunity [9]–[13]. Next to the well-established function as regulator of the complement cascade, CD55 is engaged in complement-independent processes and is hijacked by several viral and bacterial pathogens to promote cell adhesion and invasion [14], [15]. Furthermore, we demonstrated previously that CD55 is a binding partner of CD97 [16].

CD97 is a member of the EGF-TM7 family of adhesion class G protein-coupled receptors (GPCRs), abundantly expressed by virtually all immune cells [17]–[22]. Like most adhesion GPCRs, CD97 is a two-subunit molecule consisting of an extracellular α subunit that is non-covalently associated with a seven-transmembrane (TM7) β subunit [19]. At the N-terminus, CD97 possesses tandemly arranged epidermal growth factor (EGF)-like domains of which the first two interact with the N-terminal short consensus repeats (SCR) of CD55 [23]–[25]. We recently found that CD97 expression levels on leukocytes are increased significantly and reversibly in CD55 knockout mice, proving for the first time that both molecules interact *in vivo* (manuscript in preparation). The physiological consequences of the interaction between CD55 and CD97 are still poorly understood. A notable finding in mice lacking a functional CD97 gene was a raise in granulocyte numbers in the periphery [26], [27]. To explore whether this phenotype was due to abrogation of the CD97-CD55 interaction, we studied the size and functionality of the granulocyte compartments in CD55-deficient mice. We found that CD55-deficient mice, like mice that lack CD97, had increased levels of circulating granulocytes, which was due to a higher granulopoietic activity in the bone marrow. Furthermore, mice lacking CD55 were better protected against *S. pneumoniae*-induced pneumonia. Together, our data indicate a role for the interaction between CD55 and the adhesion GPCR CD97 in granulocyte homeostasis and host defense.

## Materials and Methods

### Mice

Mice deficient for CD55 (*Cd55* ^-/-^, synonym: *Daf1* ^-/-^) and CD97 (*Cd97* ^-/-^) have been generated previously by us [27], [28]. Mice lacking the receptors for C3a (*C3ar1* ^-/-^) and C5a (*C5ar1* ^-/-^) were kind gifts of Prof. Graig Gerard (Harvard Medical School, Boston, MA, USA) [29], [30]. Compound mice were created by crossing *Cd55* ^-/-^ mice with *Cd97* ^-/-^, *C3ar1* ^-/-^, and *C5ar1* ^-/-^ mice [11]. All genetically modified mice were back-crossed to C57BL/6 for at least eight generations. C57BL/6 wild-type mice were littermates or were purchased from Charles River (Maastricht, The Netherlands). Congenic mice expressed *Cd45*.*1* in the B6.SJL strain. All mice used in this study were matched for age and sex and kept under specific pathogen-free conditions.

The research described in this paper complied with the ethics guidelines of the University of Amsterdam. All experiments were approved by the Animal Care and Use Committee of the University of Amsterdam under the following project numbers: DSK35, DSK1100, DSK100738, DSK101686, and DIX100121.

### Flow cytometry

Peripheral blood was collected in heparin by heart puncture. Single cell suspensions of spleen were made by mashing the organs through a 70-µm cell strainer. Bone marrow cells were harvested from dissected femurs by flushing the bone marrow plug with phosphate buffered saline PBS/0.5% bovine serum albumin (BSA). Erythrocytes were lysed with a buffer containing 155 mM NH_4_Cl, 10 mM KHCO_3_, and 1 mM EDTA in all these cell preparations. 25 µl whole blood or 5×10^5^ splenocytes or bone marrow cells were used per staining. Nonspecific binding of monoclonal antibodies (mAbs) was blocked by adding 10% normal mouse serum and 1.25 µg/ml anti-CD16/32 (clone 2.4G2; BD Biosciences). Staining was performed with appropriately diluted PE-conjugated anti-Gr-1 or anti-Ly6G and APC-conjugated anti-CD11b (eBioscience, San Diego, CA, USA) in PBS containing 0.5% BSA for 30 min at 4°C. Flow cytometric analysis was performed using a FACSCalibur or FACSCanto (BD Biosciences) and the FlowJo software package (Tree Star, Ashland, OR, USA). Absolute numbers were calculated on basis of total cell counts measured on a CASY cell counter (Schärfe, Reutlingen, Germany) or FACSCalibur multiplied by the percentage of cells positive for a specific marker, as measured by flow cytometry.

### Demargination assay

Heparin blood samples were taken 2 days before and 30 min after an intraperitoneal (i.p.) injection of 0.25 mg/kg epinephrine [31] via vena saphena and heart puncture, respectively. Erythrocytes were lysed as described above and PBL were analyzed for cellular composition by flow cytometry.

### Assessment of apoptosis

Peripheral blood lymphocytes (PBL) were cultured in the presence of RPMI with 10% fetal calf serum for 20 h at 37°C. At 0 and 20 h, the amount of viable granulocytes was analyzed by flow cytometry with the use of Mitotracker Orange (Invitrogen, Carlsbad, CA, USA) and antibodies against CD11b and Ly6G (eBioscience).

### BrdU labeling

Bromodeoxyuridine (BrdU; Sigma-Aldrich, St. Louis, MO, USA) was given by a single i.p. injection at a dose of 5 mg/mouse [32]. Blood was obtained daily via vena saphena puncture to monitor BrdU^+^Ly6G^+^ cells. Erythrocytes were lysed as described above, and cells were stained with PE-conjugated Ly6G antibody. After fixation steps with −20°C-cold 70% ethanol (30 min on ice) and paraformaldehyde (minimum 3 h at 4°C), cells were treated with DNAse (Sigma-Aldrich) (10 min at room temperature followed by 30 min on ice) and stained intracellularly with a FITC-conjugated BrdU antibody (eBioscience). Analysis was performed by flow cytometry. Absolute numbers of BrdU-positive granulocytes were calculated on the basis of granulocyte numbers in blood and the percentage of BrdU-positive granulocytes within the Ly6G-positive gate.

### Determination of cell cycle status

2×10^6^ bone marrow cells, prepared without lysing erythrocytes, were stained for Gr-1 and subsequently fixated in −20°C-cold 70% ethanol for 30 min on ice. At least 5 min before analysis by flow cytometry, 10 µg/ml propidium iodide (PI; Sigma-Aldrich) was added to the cells. Doublets and debris were excluded from analysis by setting an appropriate FSC-SSC gate, and Gr-1^+^ cells with > 2N DNA content, measured with PI, were considered to be in G2/S phase [33].

### Pneumococcal pneumonia

Pneumonia was induced in wild-type and *Cd55* ^-/-^ mice as described previously [27]. Briefly, *S. pneumoniae* serotype 3 was obtained from American Type Culture Collection (ATCC 6303; Rockville, MD, USA). Pneumococci were grown for 6 h to mid-logarithmic phase at 37°C in 5% CO_2_ using Todd-Hewitt broth (Difco, Detroit, MI, USA), harvested by centrifugation at 1500 x g for 15 min, and washed twice in sterile isotonic saline. Bacteria were then resuspended in sterile isotonic saline at a concentration of approximately 9×10^4^ colony-forming units (CFU)/50 µl as determined by plating serial 10-fold dilutions on sheep blood agar plates. Mice were lightly anesthetized and inoculated intranasally (i.n.) with 50 µl.

At 24 h and 48 h after inoculation, mice were anesthetized and sacrificed by heart puncture. Blood was collected in EDTA-containing tubes, and lungs and spleen were harvested in sterile saline. One lung lobe was fixed in 10% formalin and embedded in paraffin. 4-µm sections were stained with hematoxylin and eosin (H&E) and analyzed by a pathologist who was blinded for groups. The other lung lobe and spleen were homogenized at 4°C in four volumes of sterile saline using a tissue homogenizer (BioSpec Products, Bartlesville, OK, USA). CFU were determined from serial dilutions of lung and spleen homogenates and blood, plated on blood agar plates and incubated at 37°C at 5% CO_2_ for 16 h before colonies were counted. For cytokine and chemokine measurements, lung homogenates were diluted 1:2 in lysis buffer containing 300 mM NaCl, 30 mM Tris, 2 mM MgCl_2_, 2 mM CaCl_2,_ 1% Triton X-100, and pepstatin A, leupeptin, and aprotinin (all 20 ng/ml; pH 7.4) and incubated at 4°C for 30 min. Homogenates were centrifuged at 1500 x g at 4°C for 15 min, and supernatants were stored at -20°C until assays were performed. Cytokines (TNF, IL-1β) and chemokines (KC, macrophage-inflammatory protein-2 (MIP-2)) were measured using specific ELISA (R&D Systems, Minneapolis, MN, USA) according to the manufacturer's instructions. Myeloperoxidase levels were quantified by MPO ELISA (HBt, Uden, The Netherlands) according to the manufacturer's protocol. In a separate group of mice, survival was monitored.

### Thioglycollate-induced peritonitis

Sterile peritonitis was induced by i.p. injection of 1 ml 4% aged thioglycollate broth (Sigma-Aldrich). After 4 h, peritoneal lavages were performed with 4 ml ice-cold 2 mM EDTA in PBS. Peritoneal cells were counted on a CASY cell counter (Schärfe, Reutlingen, Germany) and analyzed for cellular composition by flow cytometry.

### Statistical analysis

Differences between groups were calculated by unpaired t-test or Mann-Whitney *U* test. For comparison of multiple groups one-way ANOVA with Bonferroni's multiple comparison test was used. The effect of lack of CD55 on the number of BrdU-positive granulocytes in circulation was assessed by calculating the cumulative area under the curve, followed by the Wilcoxon rank-sum test. For survival analysis, Kaplan-Meier analysis followed by log rank test was performed. A two-tailed *p* value of less than 0.05 was considered to represent a significant difference.

## Results

### Mice lacking CD55 have increased levels of circulating granulocytes

**Figure 1:**
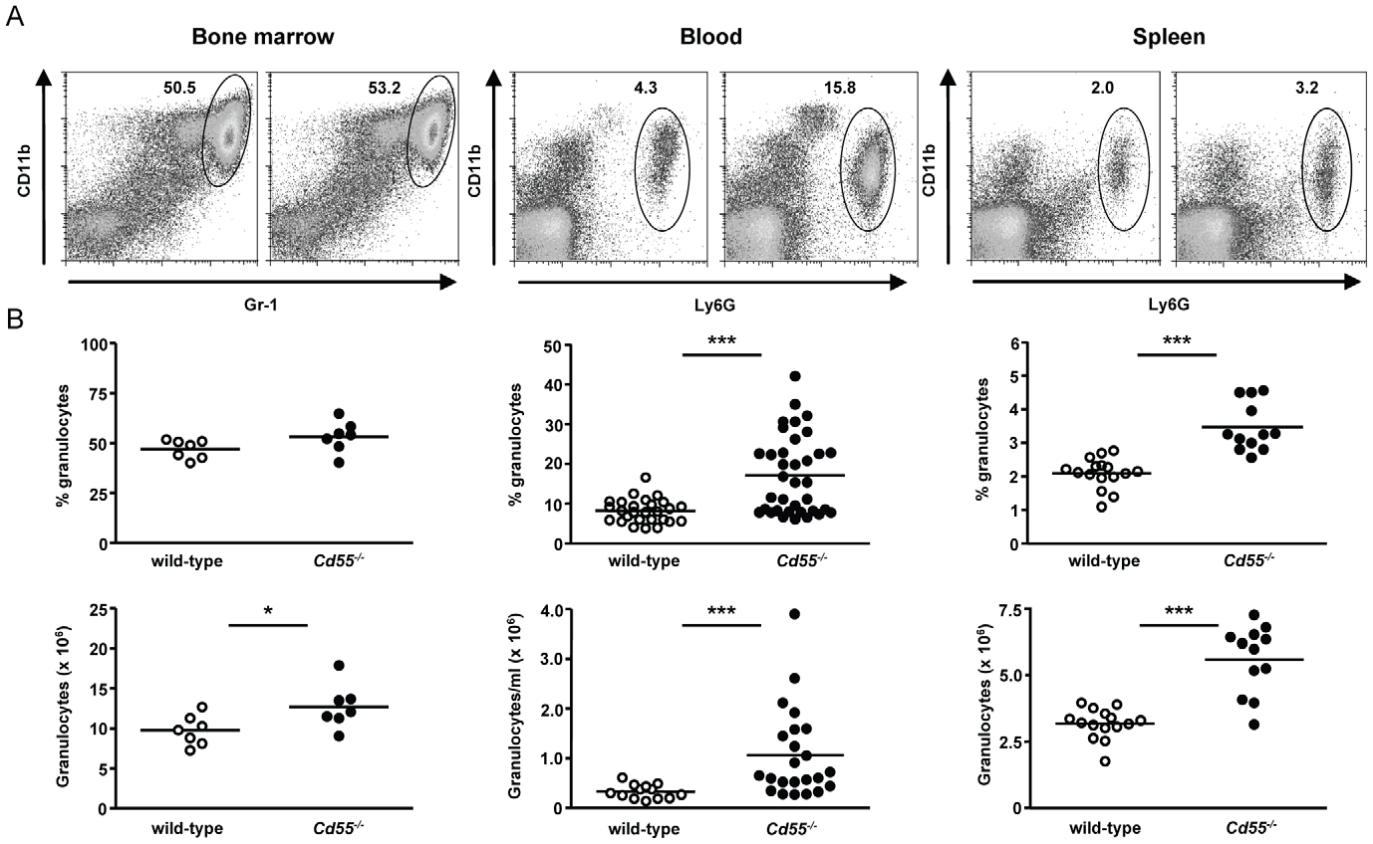
Lack of CD55 causes a mild granulocytosis. Immune cells obtained from bone marrow, blood, and spleen of wild-type and *Cd55* ^-/-^ mice were incubated with antibodies against Ly6G (or Gr-1) and CD11b and analyzed by flow cytometry. (**A**) Representative dot plots showing Gr-1 against CD11b expression on bone marrow-derived cells and Ly6G against CD11b expression on PBL and splenocytes of wild-type and *Cd55* ^-/-^ mice. (**B**) Percentages (upper panels) and total numbers (lower panels) of mature granulocytes in one femur (CD11b^+^Gr-1^high^), 1 ml blood (CD11b^+^Ly6G^+^), or in total spleen (CD11b^+^Ly6G^+^) of wild-type and *Cd55* ^-/-^ mice. Circles represent individual mice and horizontal lines indicate the mean of the percentage or absolute number of granulocytes per group (n = 7-35). *, p<0.05 and ***, p<0.0005

**Figure 2:**
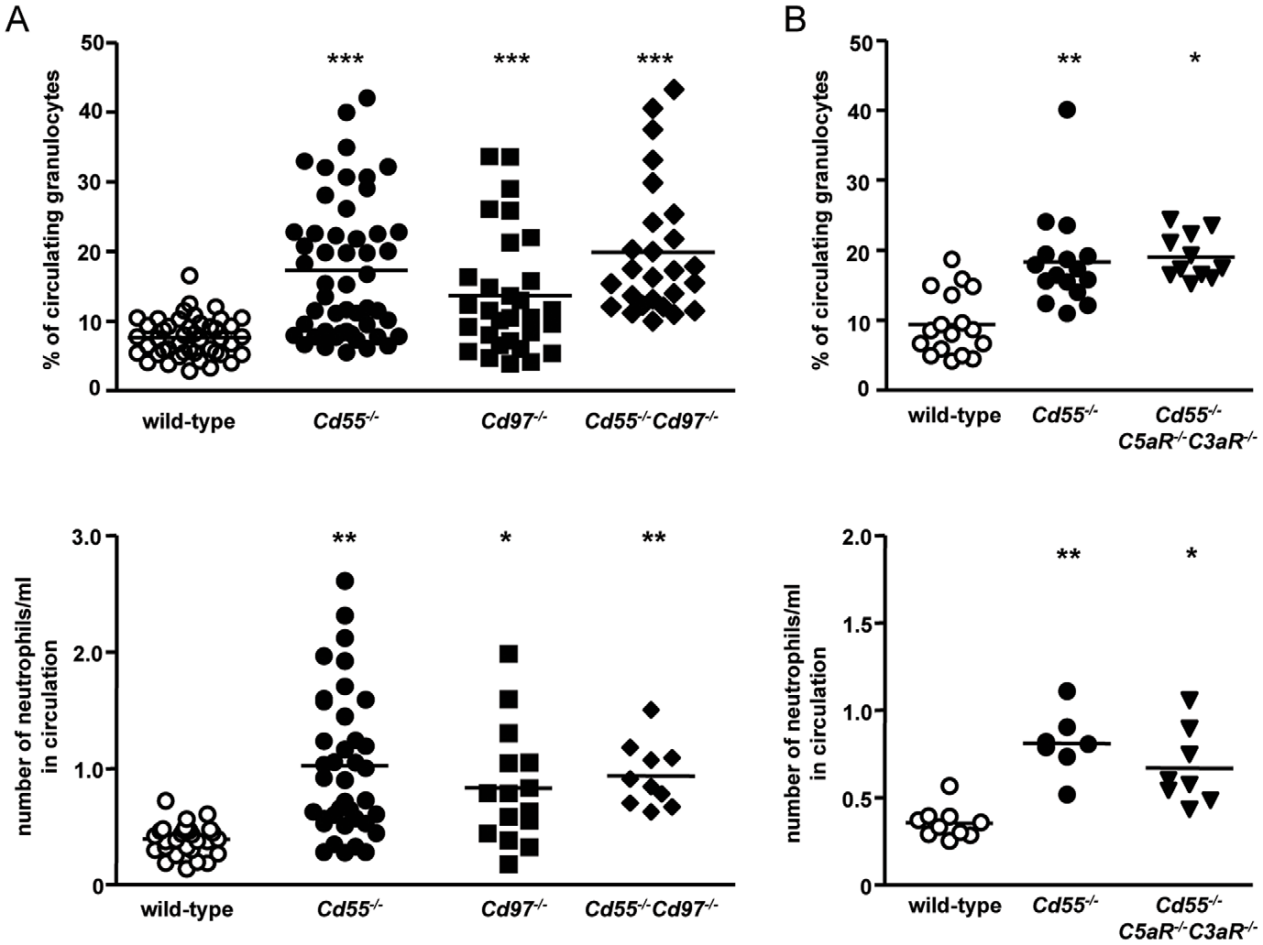
Increased granulocyte numbers in *Cd55^-/-^* mice does not result from enhanced complement activity. Immune cells obtained from blood of wild-type and knockout mice were incubated with antibodies against Ly6G and CD11b and analyzed by flow cytometry. Percentages (upper panels) and total numbers (lower panels) of granulocytes in blood of (**A**) wild-type, *Cd55* ^-/-^, *Cd97* ^-/-^, and *Cd55* ^-/-^ *Cd97* ^-/-^ mice and (**B**) wild-type, *Cd55* ^-/-^, and *Cd55* ^-/-^ *C3ar* ^-/-^ *C5ar* ^-/-^ mice. Circles represent individual mice, and horizontal lines indicate the mean of the percentage or absolute number of granulocytes per group (n = 7-38). *, p<0.05; **, p<0.005; ***, p<0.0005

CD97-deficient mice display a mild granulocytosis [26], [27]. When we analyzed the hematopoietic compartment in *Cd55* ^-/-^ mice, we found a comparable phenotype (Figure 1A+B). The percentage and absolute number of CD11b^+^Ly6G^+^ granulocytes was increased in the blood of about half and in the spleen of almost all mice lacking CD55. In bone marrow, the increase in mature granulocyte (CD11b^+^Gr-1^high^) numbers was less pronounced. There were no differences in the amount of CD11b^+^Gr-1^int^ cells, which according to Ueda et al. [33] represent immature precursors of granulocytes. Next to granulocytes, we did observe an increased amount of monocytes in the blood of mice lacking CD55; yet, this was not consistent and not clearly found in spleens of these mice. The striking similarity between *Cd55* ^-/-^ and *Cd97^-/-^* mice with respect to granulocyte numbers led us to compare them side-by-side in combination with compound mice, lacking both genes. We found a comparable increase in the percentage and absolute number of granulocytes in *Cd55* ^-/-^ and *Cd97* ^-/-^ mice that was not further increased in double knockout *Cd55^-/-^Cd97^-/-^* mice (Figure 2A).

Increased local production of C3a and C5a and subsequently increased C3a and C5a receptor signaling in *Cd55* ^-/-^ mice has been shown to be involved in T-cell survival [11]. Furthermore, C5a has been demonstrated to protect granulocytes from apoptosis [34]. In order to test whether increased C3a and C5a receptor signaling was involved in the displayed granulocytosis in *Cd55* ^-/-^ mice, we assessed circulating granulocyte numbers in *Cd55^-/-^C3ar^-/-^C5ar^-/-^* compound mice. Figure 2B shows that CD55-deficient mice still displayed granulocytosis when C3a and C5a receptor signaling was abrogated, indicating that increased granulocyte numbers in the *Cd55^-/-^* mice did not result from enhanced complement receptor activation.

### Expanded granulopoietic activity in CD55-deficient and CD97-deficient mice

**Figure 3:**
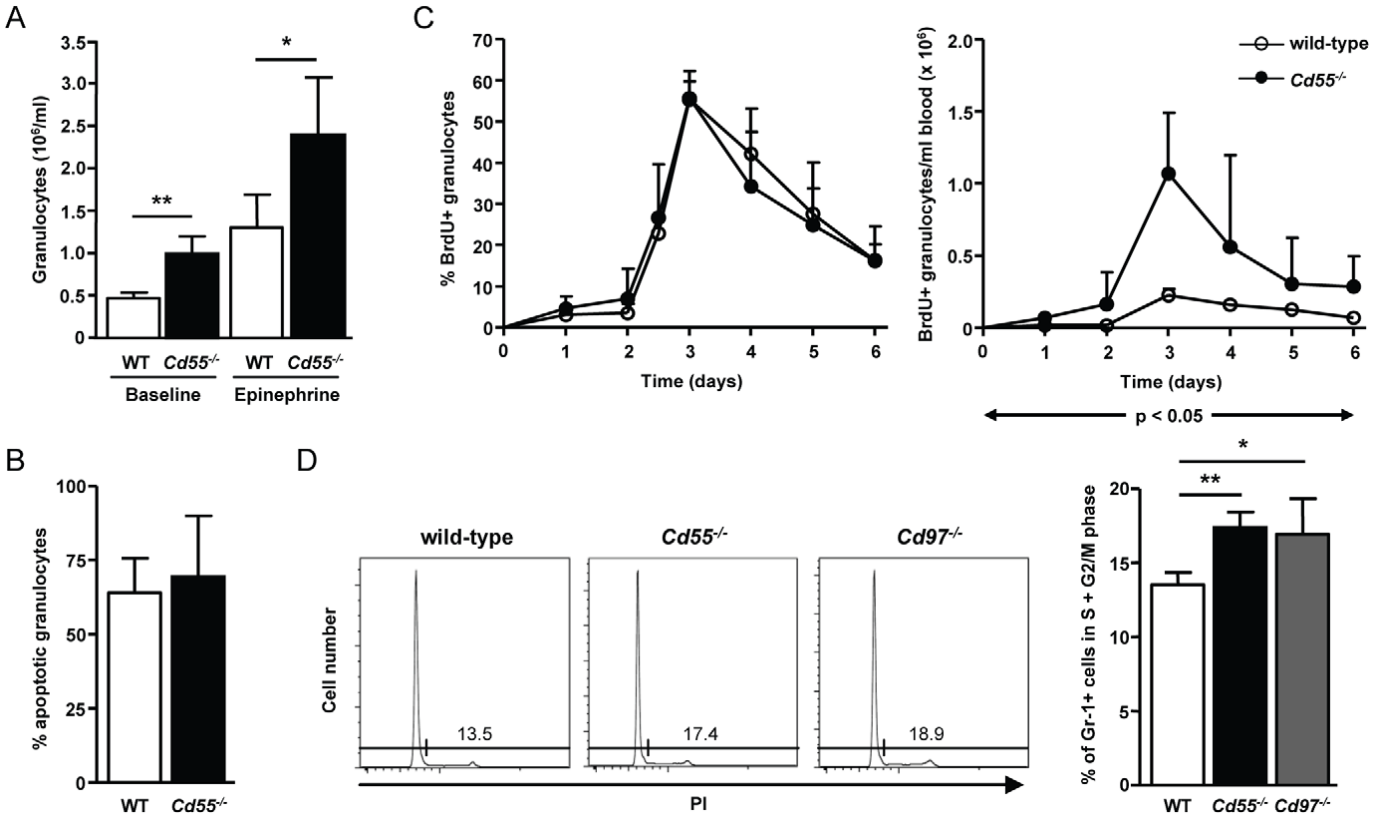
*Cd55^-/-^* and *Cd97^-/-^* mice have an increased granulopoietic capacity. (**A**) Blood was collected from wild-type and *Cd55* ^-/-^ mice two days before and 30 min after i.p. injection of 0.25 mg/kg epinephrine, and the number of granulocytes per ml blood was measured by flow cytometry. Bars and error bars represent the mean and SD of the number of granulocytes (n = 3-5). (**B**) PBL from wild-type and *Cd55* ^-/-^ mice, cultured overnight at 37°C, were stained with Mitotracker and analyzed by flow cytometry. Shown is the mean and SD of the percentage of Mitotracker-negative apoptotic granulocytes (n = 4). (**C**) Blood was collected from wild-type and *Cd55* ^-/-^ mice via vena saphena puncture at the indicated time points after a single i.p. injection of 5 mg BrdU. PBL were stained for Ly6G and BrdU and analyzed by flow cytometry (left panel). The mean and SD of the percentages (left panel) and absolute numbers (right panel) of BrdU-positive granulocytes are depicted (n = 3–5). (**D**) Wild-type, *Cd55* ^-/-^, and *Cd97^-/-^* bone marrow cells were harvested and analyzed by flow cytometry for dividing cells. To the left, representative histograms showing the percentage of granulocytes with > 2N DNA content in wild-type, *Cd55* ^-/-^, and *Cd97* ^-/-^ mice are provided. To the right, the mean and SD of the percentage of granulocytes with > 2N DNA content is depicted (n = 3–4). One of two comparable experiments is shown. *, p<0.05 and **, p<0.005

In order to obtain insight into the mechanism behind the observed granulocytosis, we started out by exploring the total population of mature peripheral granulocytes. These can exist as freely circulating cells as well as reversibly adhered cells to the vascular endothelium [35]. The latter so called marginated cells can be released into circulation by epinephrine injection, making them accessible for blood sampling [31]. We found that in response to i.p. application of epinephrine, granulocyte numbers in wild-type mice increased almost 3-fold from 4.6×10^5^/ml to 13.1×10^5^/ml. *Cd55* ^-/-^ mice exhibited a similar increase (10.0×10^5^/ml to 23.8×10^5^/ml), resulting in granulocyte counts that again were significantly higher compared to wild-type mice (Figure 3A). The normal epinephrine response seen in mice lacking CD55 suggested that the displayed granulocytosis in these mice is not caused by a defect in margination of granulocytes.

Pre-mature mobilization from bone marrow would be another possibility to explain the increased number of granulocytes in circulation [35]. However, when we analyzed blood smears of *Cd55* ^-/-^ mice, we found that almost all granulocytes had a mature, segmented phenotype, and no differences were found between *Cd55* ^-/-^ granulocytes compared to wild-type cells (data not shown).

We next tested the possibility of an increased survival of granulocytes in the periphery in *Cd55* ^-/-^ mice. We started with the assessment of granulocyte apoptosis *ex vivo* by culturing PBL for 20 h, followed by measuring survival rates using Mitotracker. Both wild-type and *Cd55* ^-/-^ granulocytes displayed a considerable amount of apoptosis, which was not different between the two mouse strains (Figure 3B). Granulocytes are short-lived cells with a half-life in circulation of less than one day under normal conditions [35]; and although *Cd55* ^-/-^ granulocytes did not display an altered apoptosis rate *ex vivo*, it could be possible that they are not cleared from circulation as efficient as in wild-type mice. To assess this possibility, we studied the life span of circulating granulocytes *in vivo*. To this end, dividing granulocytes in the bone marrow were pulse-labeled by a single i.p. injection of BrdU [32], and their kinetics in circulation was determined by measuring Ly6G-positive cells that had incorporated BrdU. We found a comparable transit time for BrdU-positive granulocytes to appear in circulation in wild-type and *Cd55* ^-/-^ mice with a peak percentage at 72 h (Figure 3C, left panel). Also the disappearance of labeled cells from blood was not changed in *Cd55* ^-/-^ mice (Figure 3C, left panel). However, the numbers of BrdU-positive granulocytes in circulation in *Cd55* ^-/-^ mice were significantly higher throughout the duration of the experiment (Figure 3C, right panel). We concluded that despite a normal life span in circulation, the amount of granulocytes produced and released from the bone marrow was significantly increased in *Cd55* ^-/-^ mice. Interestingly, Wang et al. showed that CD97-deficient mice had no altered apoptotic rate but they observed an increased response to daily G-CSF injections, also suggesting greater granulopoietic production [26].

To corroborate the hypothesis that both CD55-deficient and CD97-deficient mice had an increased granulopoietic activity, we studied the proliferative activity of Gr-1-positive cells in the bone marrow by measuring their DNA content [33]. *Cd55* ^-/-^ mice displayed an increased percentage of cells that were in S or G2/M phase of the cell cycle as compared to wild-type mice (Figure 3D). Of note, side-by-side comparison revealed a similar increase in the percentage of dividing Gr-1-positive cells in the bone marrow in *Cd97^-/-^* mice (Figure 3D). Together, these data suggest that the granulocytosis in the periphery of mice lacking either CD55 or CD97 is caused by an increased granulopoiesis.

### Augmented protection against pneumococcal pneumonia in CD55-deficient mice

**Figure 4:**
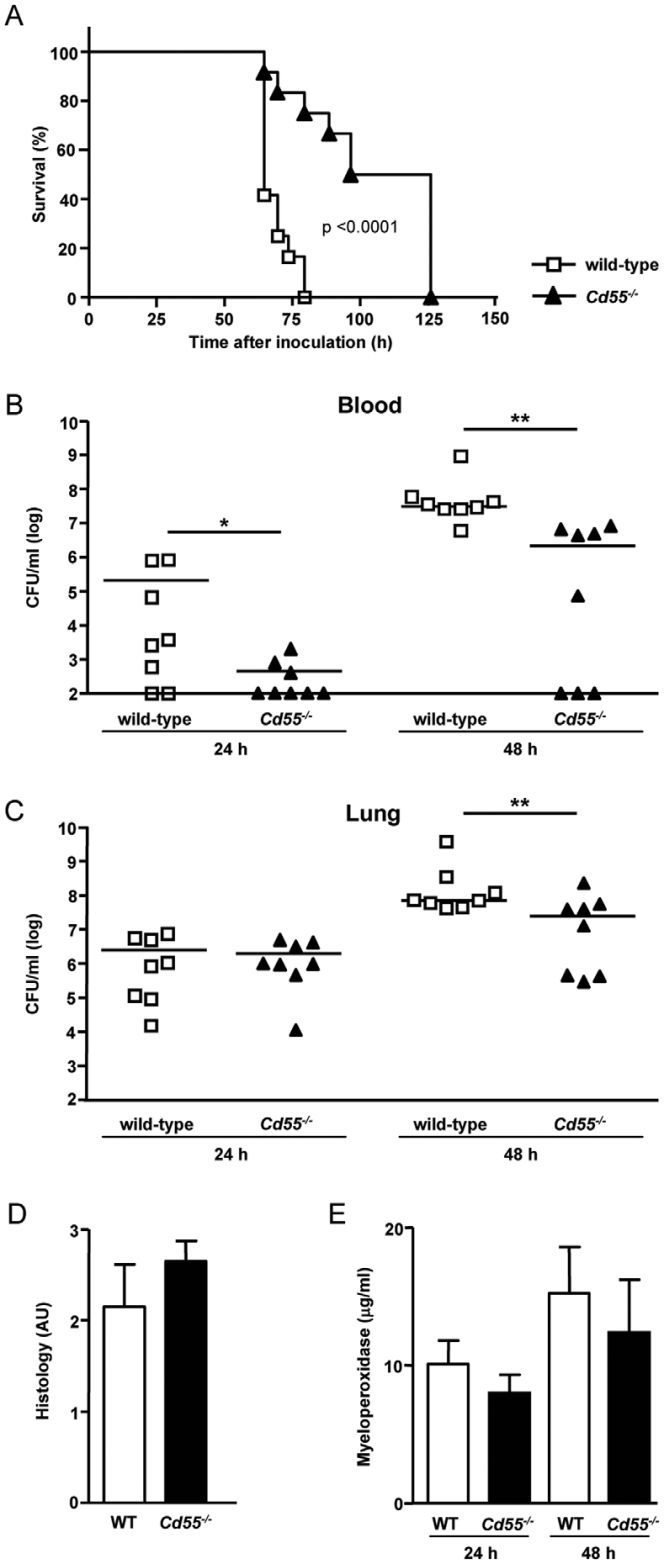
*Cd55^-/-^* mice have an increased resistance against *Streptococcal pneumoniae* infection. Wild-type and *Cd55* ^-/-^ mice were i.n. infected with 8×10^4^ CFU *S. pneumoniae* (**A**) Survival of 12 mice per group. (**B,C**) Outgrowth of pneumococcal bacteria in blood (**B**) and lungs (**C**) of wild-type and *Cd55^-/-^* mice at 24 h or 48 h after infection. Squares and triangles represent individual mice. Bars indicate median bacterial counts (n = 8 in all groups). (**D**) Total histopathological scores of lungs obtained from wild-type and *Cd55^-/-^* mice at 48 h after infection. Indicated is the mean ± SEM of six parameters (pneumonia, bronchitis, pleuritis, interstitial inflammation, oedema, and endothelialitis) (n = 8) in arbitrary units (0 to 4) (**E**) Concentration of myeloperoxidase in lung homogenates of wild-type and *Cd55^-/-^* mice at 24 h and 48 h after infection. Indicated is the mean ± SEM of the concentration of myeloperoxidase in lung homogenates (n = 8 in all groups). *, p<0.05 and **, p<0.005

To determine possible consequences of CD55 deficiency for anti-bacterial host defense, mice were inoculated with a lethal dose of *S. pneumonia* (9×10^4^ CFU) and monitored over time. While wild-type mice all died at day 3 after infection (median survival time  =  64.5 h), *Cd55* ^-/-^ lived up till day 5 after infection (median survival time  =  111.3 h) (Figure 4A). To obtain insight in how *Cd55* ^-/-^ mice were protected against lethality during pneumococcal pneumonia, we examined bacterial loads in the lungs and blood 24 h and 48 h after infection (Figure 4B and C). Most notably, at these time points, *Cd55* ^-/-^ mice had significantly lower bacterial loads in blood. Of the wild-type animals, 75% had positive blood cultures, whereas only 37.5% of the *Cd55* ^-/-^ blood cultures were positive 24 h after infection. At 48 h, all wild-type mice showed positive blood cultures while in the *Cd55* ^-/-^ group, only 62.5% showed bacterial outgrowth (Figure 4B). Furthermore, at 48 h, *Cd55* ^-/-^ mice had slightly lower bacterial loads in their lungs compared to wild-types mice (Figure 4C).

Histopathological examination of lung sections showed no differences in lung inflammation between wild-type and *Cd55* ^-/-^ animals at 24 h (data not shown) and 48 h after infection (Figure 4D). Also, the local concentration of the pro-inflammatory cytokines TNFα and IL-1β and the chemokines KC and MIP-2 was not different between wild-type and *Cd55* ^-/-^ mice at 24 h and 48 h after infection (data not shown). Finally, the amount of phagocytic cells present in infected lungs increased over time, but no difference was found between wild-type and *Cd55* ^-/-^ mice as determined by the amount of myeloperoxidase present in lung homogenates (Figure 4E). To further substantiate the point that migration of *Cd55* ^-/-^ granulocytes to sites of inflammation is normal, we induced sterile peritonitis with 4% thioglycollate in wild-type and *Cd55* ^-/-^ mice. Also in this sterile model of inflammation, we saw comparable accumulation of granulocytes in wild-type and *Cd55* ^-/-^ mice (absolute number of granulocytes in peritoneal exudate  =  17.4×10^6^±2.0 and 17.0×10^6^±4.0, respectively).

To test the possibility that *Cd55* ^-/-^ granulocytes were functionally more competent, we examined the production of reactive oxygen species (ROS) upon various stimuli. PMA, zymosan, serum-treated zymosan, or PAF/fMLP was used to stimulate granulocytes derived from bone marrow of wild-type and *Cd55* ^-/-^ mice. No differences were found with respect to the amount of ROS produced, the time to produce it, or the percentage of granulocytes that produced ROS in response to stimulation (data not shown). We concluded that a better protection from bacteremia due to increased granulocyte numbers rather than qualitative improvement of immunity in the lungs likely reduced lethality from *S. pneumonia* in *Cd55* ^-/-^ mice.

## Discussion

We here provide evidence for a novel role of the complement control protein CD55 in granulocyte homeostasis and anti-bacterial host defense. CD55-deficient mice display increased numbers of circulating granulocytes. On average, we found twice as many granulocytes in the blood stream, the marginated pool, and the spleen of *Cd55* ^-/-^ compared to wild-type mice. This mild granulocytosis clearly differs from the marked changes in mice that lack leukocyte adhesion molecules [35]. A comparable phenotype has recently been reported in two independently generated CD97-deficient mice [26], [27]. The Kelly laboratory and we found that mice lacking CD97 have about two-fold increased granulocyte numbers in blood and spleen in the steady state. This phenotype was not the result of enhanced bone marrow emigration to the blood neither of reduced clearance of circulating cells. *Cd97^-/-^* mice showed a greater response to daily G-CSF treatment, which induces granulopoiesis in the bone marrow [26]. In line with this observation, we here demonstrate increased numbers of Gr-1-positive cells in cell cycle in the bone marrow of both CD55-deficient mice and CD97-deficient mice, indicating a higher granulopoietic activity in both strains.

Several pieces of evidence suggest that the role of CD55 in granulocyte homeostasis relates to its interaction with CD97. Firstly, the granulocytosis in both mouse strains is highly comparable and not further accelerated in compound mice. Secondly, mice lacking CD55 and the receptors for C3a and C5a had granulocyte numbers comparable to CD55-deficient mice, excluding a causal link with exaggerated complement receptor activation in the absence of CD55. Thirdly, as discussed above, both *Cd55^-/-^* and *Cd97^-/-^* mice displayed enhanced granulopoietic activity. These data suggest that both molecules through their interaction in bone marrow jointly affect granulocyte homeostasis. How this occurs at the molecular level is currently unknown. Both CD55 and CD97 are differentially expressed during the development of hematopoietic progenitor cells [36], [37]; moreover, CD55 is present on stromal cells and can be deposited in the extracellular matrix [1], [38]. Thus, the interaction between CD55 and CD97 can be involved in various contacts between hematopoietic progenitors and stromal cells. This concept would fit with recent data suggesting that adhesion GPCRs by facilitating cell and matrix contacts contribute to the proper positioning of developing cells in a variety of organ systems [39]–[41].

Granulocytes are crucially involved in anti-bacterial host defense. To test whether increased granulocyte number exaggerate the innate response in CD55-deficient mice, we applied an acute model of pneumococcal pneumonia. In response to a lethal dose of *S. pneumonia*, *Cd55^-/-^* mice displayed a better clearance of bacteria from blood and lung correlating with an extended survival compared to wild-type mice. Increased complement activation in the absence of CD55 may ameliorate this exaggerated response and might explain why a similar protective effect was not found previously by us in *Cd97^-/-^* mice [27]. However, Kelly and colleagues reported that *Cd97^-/-^* mice are more resistant to *Listeria monocytogenes*, correlated with a more robust accumulation of granulocytes in the blood and the liver of infected mice [26].

Our study strengthens the view that the interaction with CD97 is a physiologically relevant, complement-independent activity of CD55. Further support for the idea was obtained in a recent study, showing a comparable level of protection from experimental arthritis in *Cd55^-/-^* and *Cd97^-/-^* mice, which might relate to a role of both molecules in the retention of leukocytes in the inflamed synovium [42] Of note, CD55 is the first binding partner that is linked functionally to an adhesion GPCR *in vivo* [43].
